# *EXT1* and *EXT2* Variants in 22 Chinese Families With Multiple Osteochondromas: Seven New Variants and Potentiation of Preimplantation Genetic Testing and Prenatal Diagnosis

**DOI:** 10.3389/fgene.2020.607838

**Published:** 2020-12-22

**Authors:** Ye Wang, Liangying Zhong, Yan Xu, Lei Ding, Yuanjun Ji, Sacha Schutz, Claude Férec, David N. Cooper, Caixia Xu, Jian-Min Chen, Yanmin Luo

**Affiliations:** ^1^Fetal Medicine Center, Department of Obstetrics and Gynecology, The First Affiliated Hospital, Sun Yat-sen University, Guangzhou, China; ^2^Department of Laboratory Medicine, The First Affiliated Hospital, Sun Yat-sen University, Guangzhou, China; ^3^Reproductive Medicine Center, The First Affiliated Hospital, Sun Yat-sen University, Guangzhou, China; ^4^Guangdong Provincial Key Laboratory of Reproductive Medicine, Guangzhou, China; ^5^Department of Radiology, The First Affiliated Hospital, Sun Yat-sen University, Guangzhou, China; ^6^Inserm, Univ Brest, EFS, UMR 1078, GGB, Brest, France; ^7^CHRU Brest, Brest, France; ^8^School of Medicine, Institute of Medical Genetics, Cardiff University, Cardiff, United Kingdom; ^9^Research Center for Translational Medicine, The First Affiliated Hospital, Sun Yat-sen University, Guangzhou, China

**Keywords:** *EXT1* gene, *EXT2* gene, multiple osteochondromas, pathogenic variant, preimplantation genetic testing, prenatal diagnosis

## Abstract

Multiple osteochondromas (MO), the most common type of benign bone tumor, is an autosomal dominant skeletal disorder characterized by multiple cartilage-capped bony protuberances. In most cases, *EXT1* and *EXT2*, which encode glycosyltransferases involved in the biosynthesis of heparan sulfate, are the genes responsible. Here we describe the clinical, phenotypic and genetic characterization of MO in 22 unrelated Chinese families involving a total of 60 patients. Variant detection was performed by means of a battery of different techniques including Sanger sequencing and whole-exome sequencing (WES). The pathogenicity of the missense and splicing variants was explored by means of *in silico* prediction algorithms. Sixteen unique pathogenic variants, including 10 in the *EXT1* gene and 6 in the *EXT2* gene, were identified in 18 (82%) of the 22 families. Fourteen (88%) of the 16 variants were predicted to give rise to truncated proteins whereas the remaining two were missense. Seven variants were newly described here, further expanding the spectrum of MO-causing variants in the *EXT1* and *EXT2* genes. More importantly, the identification of causative variants allowed us to provide genetic counseling to 8 MO patients in terms either of preimplantation genetic testing (PGT) or prenatal diagnosis, thereby preventing the reoccurrence of MO in the corresponding families. This study is the first to report the successful implementation of PGT in MO families and describes the largest number of subjects undergoing prenatal diagnosis to date.

## Introduction

Multiple osteochondromas (MO) (OMIM 133700-133701), also known as hereditary multiple exostoses (HME) or osteocartilaginous exostosis, is an autosomal dominant skeletal disorder characterized by the formation of benign cartilage-capped bony protuberances, typically arising on the metaphyses of long tubular bones (Bovee, 2008). The malformation of legs, forearms and hands is a frequent manifestation (Matsubara et al., 2006). The prevalence of MO is estimated to be 1/50,000 (Wicklund et al., 1995) and would appear to be higher in males (male-to-female ratio, 1.5:1) (D’Arienzo et al., 2019), making it one of the most frequent causes of skeletal dysplasia. MO is characterized by significant inter- and intra-familial phenotypic heterogeneity, including variation in the number and size of osteochondromas, the number and location of the bones involved, and the degree of the deformities arising (Peterson, 1989). Osteochondromas are rarely present at birth, but rather appear and grow during the first decade of life, finally ceasing to grow when the growth plates close at the end of puberty. Approximately 62% of MO patients have a positive family history, with nearly complete penetrance (Schmale et al., 1994).

Benign as the osteochondromas are, they can lead to several complications. By exerting pressure on neighboring tissues, osteochondromas cause pain, nerve compression, and other conditions (Pedrini et al., 2005). Skeletal malformations are also observed in MO patients with limb length inequalities, restricted range of joint motion and short stature (Porter et al., 2004). At present there is no etiological treatment, so surgery may be the only way to correct the severe deformities. Although MO does not in itself affect life expectancy, malignant transformation to a chondrosarcoma occurs in 1–5% of cases (Peterson, 1989; Pedrini et al., 2011).

MO is genetically heterogeneous. To date, three loci for MO have been mapped and two genes, *EXT1* (Entrez gene ID: 2131, alias: Exostosin1) and *EXT2* (Entrez gene ID: 2132, alias: Exostosin2), have been cloned (Ahn et al., 1995; Stickens et al., 1996). *EXT1* consists of 11 exons and spans ∼312 kb at 8q24 (Ludecke et al., 1997), while *EXT2* comprises 16 exons and is located at 11p11.2, spanning ∼150 kb (Clines et al., 1997). The genes belong to the *EXT* multigene family, are ubiquitously expressed and act as tumor suppressors. All members of this family encode proteins that are involved in the adhesion and/or polymerization of heparin sulfate (HS) chains at HS proteoglycans (HSPG’s) (Lind et al., 1998; McCormick et al., 1998; Busse et al., 2007). *EXT1* and *EXT2* function as glycosyltransferases that participate in the biosynthesis of heparin sulfate (HS) to modify proteoglycans. HS proteoglycans, synthesized by chondrocytes and secreted to the extracellular matrix of the growth plate, play critical roles in growth plate signaling and remodeling.

To date, hundreds of *EXT1* and *EXT2* variants have been registered in the Human Gene Mutation Database (HGMD)^1^ (Stenson et al., 2020) as well as in the *EXT1*^2^ and *EXT2*^3^ locus-specific mutation databases. Most of the known pathogenic variants cause truncation of the encoded proteins, e.g., frameshift, non-sense, and splice site mutations; pathogenic missense variants are uncommon (Santos et al., 2018; Fusco et al., 2019). The application, more recently, of structural variant-detection techniques such as multiplex ligation-dependent probe amplification (MLPA) and microarray analysis has served to increase the variant detection rate from 70 to 85% (Li et al., 2018; Fusco et al., 2019). In the present study, we report the genetic findings from 22 Chinese MO families together with the successful use of this information to potentiate preimplantation genetic testing (PGT) and prenatal diagnosis in 8 MO families.

## Materials and Methods

### Ethics Statement

This study was approved by the Ethics Committee of Sun Yat-sen University. All participants (or parents/guardians when the participants were under the age of 18) gave their informed consent.

### Subjects

Sixty MO patients (41 male) from 22 unrelated Chinese families participated in this study. All probands were referred to the First Affiliated Hospital of Sun Yat-sen University from January 2011 to September 2018. A diagnosis of MO was made on clinical, radiographic and laboratory findings, in particular the existence of at least two exostoses at the juxta-epiphysial regions of the long bones (Legeai-Mallet et al., 1997).

### Sanger Sequencing

Genomic DNA was extracted from peripheral blood using the QiAamp DNA Blood Mini Kit (Qiagen, Hilden, Germany). Primers were designed by Primer Premier 3.0^4^ to amplify each exon and intron/exon junction of the *EXT1* and *EXT2* genes (primer sequences and PCR conditions are available upon request). The resulting PCR products were sequenced on the ABI 3730XL DNA Genetic Analyzer (Applied Biosystems, Foster City, CA), using the ABI BigDye Terminator v3.1 Cycle Sequencing Kit.

### Whole-Exome Sequencing

Genomic DNA was fragmented randomly and then purified by means of the magnetic particle method. Sequences were captured by Agilent SureSelect version 4 (Agilent Technologies, Santa Clara, CA) according to the manufacturer’s protocols. The DNA libraries, after enrichment and purification, were sequenced on the NextSeq500 sequencer according to the manufacturer’s instructions (Illumina, San Diego). The sequencing reads were aligned to GRCh37.p10 using Burrows-Wheeler Aligner software (version 0.59) (Li and Durbin, 2009). Local realignment and base quality recalibration of the Burrows-Wheeler aligned reads were then performed using the GATK IndelRealigner^5^ and GATK BaseRecalibrator^6^, respectively. SNVs and small indels were identified by the GATK UnifiedGenotyper^7^. Variants were annotated using the Consensus Coding Sequences Database at the National Centre for Biotechnology Information^8^.

### Multiplex Ligation-Dependent Probe Amplification, Fluorescence *in situ* Hybridization and Whole Genome Sequencing

These methods have been described in detail elsewhere (Su et al., 2015; Wang et al., 2015).

### Reference Sequences and Variant Nomenclature

NM_000127.2 and NM_207122.1 were used as *EXT1* and *EXT2* mRNA reference sequences, respectively. Variant nomenclature followed HGVS recommendations (den Dunnen et al., 2016). Variants were checked against the Human Gene Mutation Database (HGMD)^9^ as well as the *EXT1* or *EXT2* locus-specific mutation database to identify previously described lesions (as of 12 Sep 2020). For those that were newly described here, their nomenclature was verified via VarSome^10^ (Kopanos et al., 2019).

### Pathogenicity Predictions

The PP3 rule established by VarSome (Kopanos et al., 2019) was adopted to predict the pathogenicity of *EXT1* missense variants as previous described (Gueguen et al., 2020). The PP3 verdict was based upon the combined consideration of predictions from a dozen *in silico* programs including BayesDel_addAF, DEOGEN2, FATHMM-MKL, M-CAP, MVP, MutationAssessor, MutationTaster, PrimateAI, REVEL and SIFT. *In silico* splicing prediction was performed by means of Alamut^®^ Visual v.2.11 rev. 0^11^ (Interactive Biosoftware, Rouen, France) and SpliceAI. Alamut includes five prediction algorithms, namely SpliceSiteFinder-like, MaxEntScan, NNSPLICE, GeneSplicer, and Human Splicing Finder. SpliceAI is a recently developed deep residual neural network for splicing prediction (Jaganathan et al., 2019).

### Structural Modeling of EXT1

Structural modeling of human EXT1 was carried out using the Robetta server^12^. Model minimization was carried out using Discovery Studio 3.5 (Accelrys, San Diego, California). The refined model was validated by the VERIFY-3D program in Discovery Studio 3.5. The model figure was prepared using PyMol (DeLano, 2002).

### Preimplantation Genetic Testing (PGT)

The oocyte retrieval, embryo culture, and biopsy methods were used as previously described (Liu et al., 2020). The biopsied trophectoderm cells transferred to phosphate buffer saline were subjected to whole-genome amplification (WGA) using the Multiple Displacement Amplification approach (REPLI-g Single Cell Kit, QIAGEN Inc.).

Three couples were offered counseling and information on different PGT platforms. SNP microarrays were used for Family 4-II-3 and Family 13-II-5. The peripheral blood DNA of parents and reference samples (abortion with *EXT1* mutation or affected parent), together with the embryo samples from the WGA, were analyzed using HumanKaryomap-12 BeadChips (Illumina, San Diego, CA, United States) according to the manufacturer’s instructions. The scanning results were analyzed using BlueFuse Multi V4.5 software provided by Illumina. The haplotypes in each pedigree was performed in the *EXT1* regions as well as 2 Mb upstream and downstream of this gene. At the same time, the B allele and Log R Ratio data were obtained from the scanning results produced by Illumina Genome studio 2.0 software for aneuploidy screening. Next-generation sequencing (NGS) platforms were used for the two PGT cycle in Family 5-II-3. The *EXT1* region was selected as the target region. A total of 31 high density and closely linked SNPs mapping within 2 Mb upstream of the gene and 33 linked SNPs mapping within 2 Mb downstream of the gene, were selected. Following mixing with corresponding PCR amplicons of the SNPs and mutations, the WGA products were used for the library construction of NGS. DNA purification, cDNA library construction and sequencing using the Illumina MiSeqDX were performed sequentially. Haplotypes were established by selection of several informative SNPs. The mutations in all samples were subjected to Sanger sequencing for verification. For PGT-A (PTG for aneuploidies), the VeriSeq PGS-MiSeq kit (Illumina) was used to prepare the NGS libraries. The MiSeq Reagent Kit v3-PGS (Illumina, San Diego, CA, United States) was used to perform dual-index 36-bp read sequencing according to the VeriSeq PGS recipe. MiSeq Reporter software (Illumina, San Diego, CA, United States) was used to make on-board secondary data analysis (Fiorentino et al., 2014). Detection and classification of aneuploidies was determined by copy number variation (CNV) values. The frozen-thawed embryos without *EXT1* mutations were transferred as previously described (Liu et al., 2020).

## Results

### Clinical Features of the Patients

**FIGURE 1:**
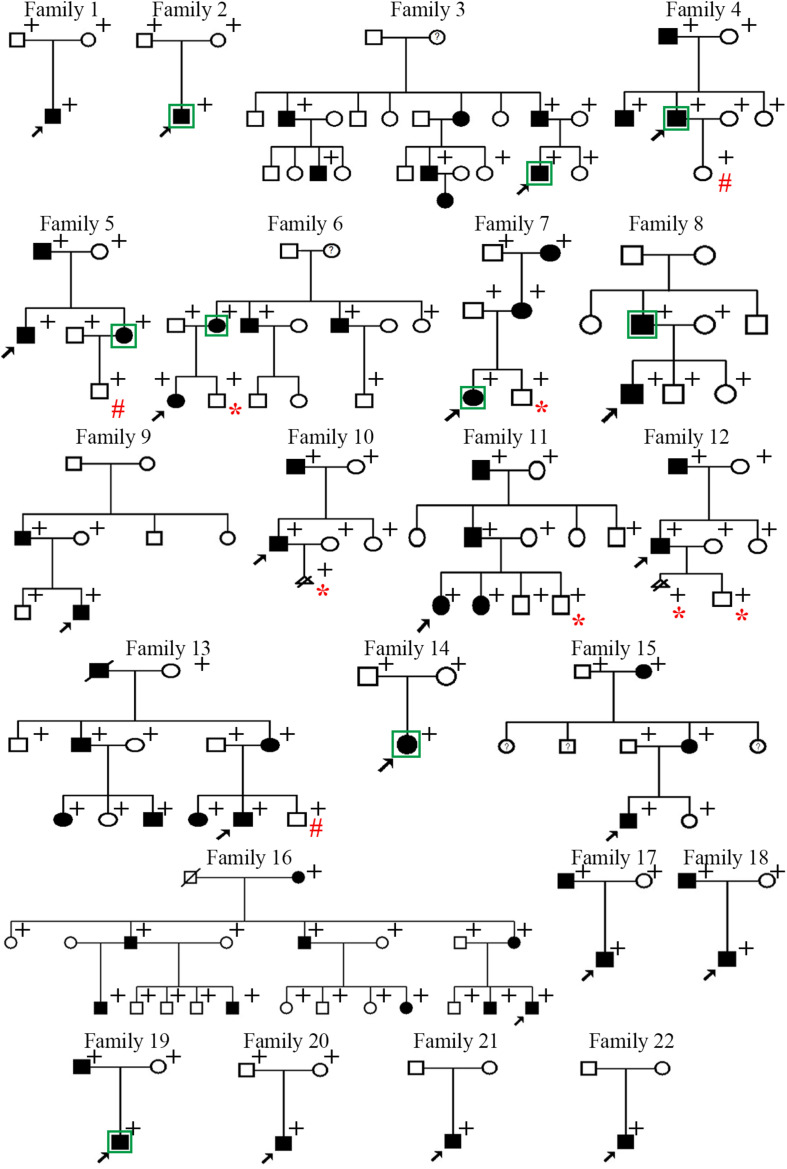
Pedigrees of the 22 Chinese families with multiple osteochondromas. Filled squares or circles denote patients. Open symbols denote clinically unaffected family members. Circles with question marks denote members with questionable clinical features and no imaging tests. Arrows indicate probands. + identifies those subjects subjected to genetic analysis. In Families 1–18, all patients subjected to genetic analysis harbored the variant of interest whereas the clinically healthy subjects did not. In Families 19–22, no pathogenic variants were found in any of the individuals subjected to genetic analysis. Those individuals who were born healthy through the preimplantation genetic testing are denoted by # whereas those who directly received prenatal diagnosis as a fetus are denoted by *. Subjects analyzed by WES were indicated by squares.

**FIGURE 2:**
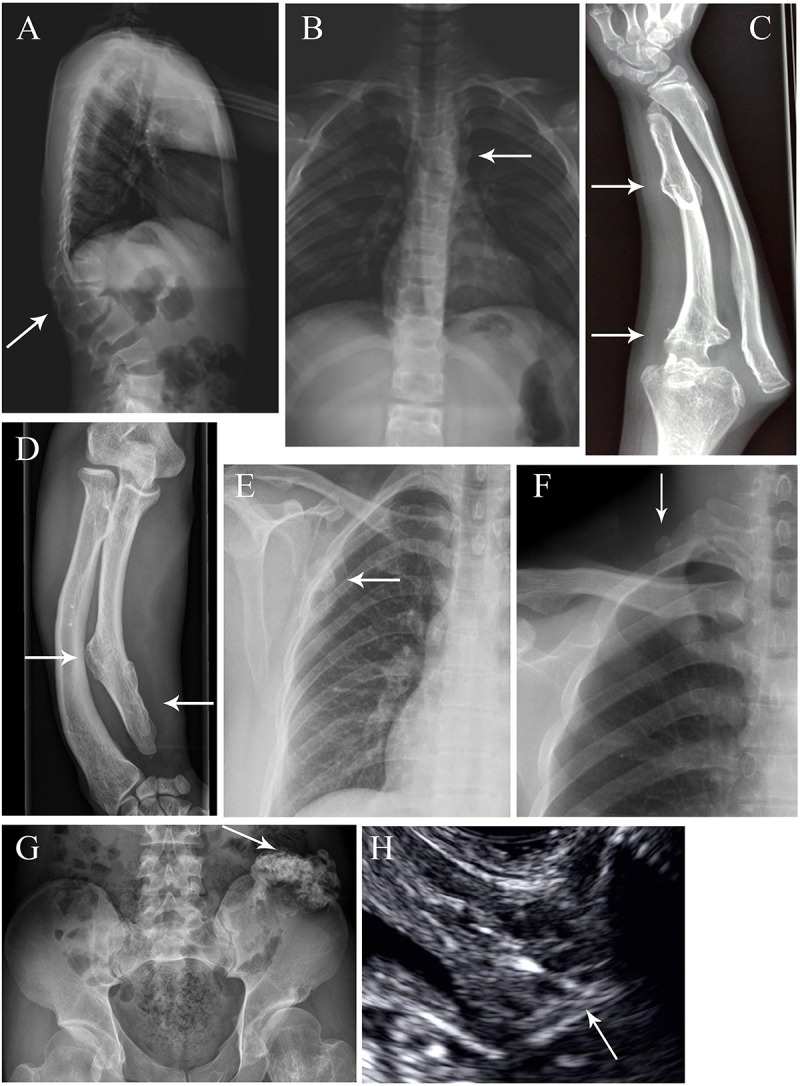
Special exostosis lesions and complications in some affected MO individuals. **(A,B)** Radiographs of Family 13 member III-5 as anteroposterior and lateral views, revealing the scoliosis, thoracolumbar kyphosis and vertebral fusion (arrow). **(C)** Right forearm of Family 11 member III-1, showing exostosis in the ulna and dislocation of the elbow joint (arrow). **(D)** Right forearm of Family 4 member II-2, showing exostosis in the ulna and bowed forearm conferring dislocation of radioulnar joint (arrow). **(E)** Radiograph of Family 7 member III-1, revealing the exostoses in the scapula (arrow). **(F)** Radiograph of Family 4 member II-2, showing the exostosis at the right side of the first rib (arrow). **(G)** Pelvic radiograph of Family 4 member II-2, displaying exostosis on the left side of the ilium (arrow). **(H)** Ultrasound images of Family 7 member III-1, showing the absence of a uterus (arrow).

The family trees of the 22 families are presented in Figure 1. Four families have been previously described [i.e., family 11 in Su et al. (2015) and families 16–18 in Wang et al. (2015)] whereas the remaining 18 families are reported for the first time. Detailed information pertaining to the clinical features exhibited was available for 36 patients, and is summarized in Table 1. Specifically, in the informative patients, osteochondroma/exostoses generally occurred in the long bones of the upper and lower limbs; chest bones (rib, sternum, scapulae, claviculae) [27.8% (10/36)] and hip (ilium, pubis, ischium) [11.1% (4/36)] were the next most commonly affected sites whereas vertebral osteochondroma/exostoses were found only in a single patient (i.e., Family 15-III-1). About 25.0% (9/36) of patients had a deformity of the limbs and joints whilst only three patients had pain in one or more affected locations. A total of 12 patients (33.3%) underwent surgery to correct the deformity. Finally, malignant transformation to chondrosarcoma occurred in two patients (i.e., Family 10-II-1 and Family 14-II-1) whilst the absence of a uterus was revealed in one patient (i.e., Family 7-III-1). Some aspects of the clinical features are also shown in Figure 2.

**TABLE 1:** Clinical characteristics of the multiple osteochondromas patients in 22 families.

Patient	Sex	Age^*a*^ (years)	Location of exostoses	Other clinical phenotypes	Surgical therapy	Pain
Family 1-II-1	M	19	Femur, tibia, fibula, ulna	Forearm deformity	No	No
Family 2-II-1	M	23	Femur, tibia, fibula, ulna, radius	Shortened ulna, negatively curved arm	No	No
Family 3- III-9	M	17	Femur, tibia, fibula, ulna, radius, clavicle	Deformity of wrist joint, dislocation of acromioclavicular joint	Yes	No
Family 4-II-2	M	28	Femur, tibia, fibula, ulna, radius, ilium, pubis	Forearm deformity, dislocation of radioulnar joint	Yes	No
Family 5-II-3	F	25	Femur, tibia, fibula	No	No	No
Family 6-II-2	F	24	Femur, tibia, fibula, ulna	No	No	No
Family 7-III-1	F	17	scapula, femur	Absence of uterus	No	No
Family 8-III-1	M	14	Femur, tibia, fibula, ulna	–	No	–
Family 9-III-2	M	10	Femur, tibia, fibula, ulna, radius, humerus, scapula	No	No	No
Family 10-II-1	M	27	Radius, femur	Chondrosarcoma	Yes	Yes
Family 11-II-2	M	40	Femur, tibia, ulna, radius	No	Yes	No
Family 11-III-1	F	11	Femur, tibia, fibula, humerus, ulna, radius, ilium, pubis, ischium, clavicle, scapula, ribs, phalanx	Forearm deformity, dislocation of elbow joint	Yes	No
Family 11-III-2	F	9	scapula, clavicle, rib	No	No	No
Family 12-II-1	M	26	Femur, tibia, ulna, radius, rib	Knee joint dysfunction	No	No
Family 13-II-1	F	37	Femur, tibia, fibula	No	No	No
Family 13-III-3	M	4	tibia, fibula, ulna, radius, humerus	Scoliosis	No	No
Family 13-III-4	F	11	Femur, tibia, fibula	No	No	No
Family 13-III-5	M	8	Femur, tibia, fibula, humerus, ulna, radius, ilium, sternum, scapula, ribs	Scoliosis, thoracolumbar kyphosis, Vertebral fusion, hemivertebra deformity	Yes	No
Family 14-II-1	F	23	Femur, tibia, fibula, rib	Chondrosarcoma	Yes	Yes
Family 15-III-1	M	13	Femur, tibia, fibula, humerus, ulna, radius, ilium, pubis, vertebra	Knee joint dysfunction	Yes	No
Family 16-I-2	F	70	Femur	Hip osteoarthritis, necrosis of femoral head, and scoliosis	No	Yes
Family 16-II-3	M	48	Humerus, tibia, ulna, and radius	No	No	No
Family 16-II-5	M	45	Femur, fibula, and radius	Dislocation of radioulnar joint	No	No
Family 16-II-8	F	40	Femur and humerus	No	No	No
Family 16-III-1	M	26	Femur, tibia, fibula, humerus, ulna and radius	Forearm deformity and wrist joint dysfunction	No	No
Family 16-III-5	M	13	Femur, tibia, fibula, ulna, and radius	No	No	No
Family 16-III-9	F	7	Femur and rib	No	No	No
Family 16-III-11	M	14	Femur, tibia, fibula, humerus, ulna, and radius	No	Yes	No
Family 16-III-12	M	12	Femur, tibia, fibula, humerus, ulna, and radius	No	Yes	No
Family 17- I -1	M	45	Femur and tibia	No	Yes	No
Family 17-II-1	M	13	Femur, tibia, fibula, and phalanx	No	No	No
Family 18-II-1	M	10	Femur, ulna, radius, and scapula	No	Yes	No
Family 19-II-1	M	25	Femur, tibia, fibula	No	No	No
Family 20-II-1	M	24	Humerus, femur, tibia	No	No	No
Family 21-II-1	M	19	Femur, fibula, radius, ulna	No	No	No
Family 22-II-1	M	22	Femur, tibia, fibula	No	No	No

**^*a*^Age at the time of diagnosis.**

### Pathogenic *EXT1* and *EXT2* Variants Found in the Patients

The *EXT1* and *EXT2* variants detected in 18 of the 22 families (i.e., families 1–18) are summarized in Table 2 alongside the detection methods employed. Of the 16 unique variants, the three large duplication or deletion variants (namely, duplication of *EXT1* exons 2–8, deletion of the entire *EXT2* gene, and deletion of exons 2–8 of *EXT2*) have been previously described in Su et al. (2015) and Wang et al. (2015). Of the remaining 13 variants, 6 were already logged in HGMD and/or *EXT1* and *EXT2* locus-specific mutation databases whereas 7 were not (as of 12 September 2020). The Sanger sequencing chromatograms for the seven newly described variants (5 in *EXT1* and 2 in *EXT2*) are shown in Supplementary Figure S1. All 16 variants were either absent or present at extremely low frequency (<0.00001) in the Genome Aggregation Database (gnomAD)^13^ (Table 2). Additionally, in families (excepting the mutation-negative Families 19–22) where additional members were available for genetic analysis, the respective variants were invariably found in patients but not in healthy subjects (Figure 1).

**TABLE 2:** *EXT1* and *EXT2* variants in the 22 Chinese families with multiple osteochondromas.

Family	Gene	Location	Nucleotide change^*a*^	Protein change^*a*^	Type of mutation	Newly described here	Allele frequency in GnomAD	Family history	Methods
1	*EXT1*	Exon 1	c.368_369delinsC	p.Lys123Thrfs*13	Frame shift		–	Negative	Sanger sequencing
2	*EXT1*	Exon 1	c.917delA	p.Lys306Serfs*53	Frame shift	Yes	–	Negative	WES
3	*EXT1*	Exon 2	c.1019G > A	p.Arg340His	Missense		3.98e-6	Positive	WES
4	*EXT1*	Exon 2	c.1019G > A	p.Arg340His	Missense		3.98e-6	Positive	WES
5	*EXT1*	Exon 2	c.1049_1051delinsAATCTGATTCC	p.Ala350Glufs*12	Frame shift	Yes	–	Positive	WES
6	*EXT1*	Exon 3	c.1065C > G	p.Cys355Trp	Missense	Yes	–	Positive	WES
7	*EXT1*	Intron 4	c.1284 + 1G > C	–	Splice site	Yes	–	Positive	WES
8	*EXT1*	Exon 9	c.1808_1836delinsTCCCAGTGGGATA	p.Tyr603Ilefs*5	Frame shift	Yes	–	Positive	WES
9	*EXT1*	Exon 10	c.1942C > T	p.Gln648*	Non-sense		–	Positive	Sanger sequencing
10	*EXT1*	Exon 10	c.2034T > G	p.Tyr678*	Non-sense		–	Positive	Sanger sequencing
11	*EXT1*	Exons 2-8	g.118825104_119054767dupins CCTTTTCTTTTTCCTTCCTTCC		Structural variant		–	Positive	Sanger + MLPA + WGS
12	*EXT2*	Exon 3	c.544C > T	p.Arg182*	Non-sense		–	Positive	Sanger sequencing
13	*EXT2*	Exon 5	c.831_832delAG	p.Glu278Valfs*7	Frame shift	Yes	3.98e-6	Positive	Sanger sequencing
14	*EXT2*	Intron 7	c.1173 + 2dupT	–	Splice site	Yes	–	Negative	WES
15	*EXT2*	Exon 8	c.1188G > A	p.Trp396*	Non-sense		–	Positive	Sanger sequencing
16	*EXT2*	Whole gene	g.43936139_44438043delins GAGAAAAGCATTTGCAAA AAGTATGA	–	Structural variant		–	Positive	Sanger + FISH + MLPA + WGS
17	*EXT2*	Whole gene	g.43936139_44438043 delinsGAGAAAAGCATTTGCAA AAAGTATGA	–	Structural variant		–	Positive	Sanger + MLPA + WGS
18	*EXT2*	Exons 2–8	g.44128440_44198500 delinsTCTTG	–	Structural variant		–	Positive	Sanger + MLPA + WGS
19	Undetected							Positive	WES + MLPA
20	Undetected							Negative	Sanger sequencing + MLPA
21	Undetected							Negative	Sanger sequencing + MLPA
22	Undetected							Negative	Sanger sequencing + MLPA

**^*a*^HGVS recommended nomenclature (www.hgvs.org/mutnomen/) was used to describe sequence variations. For the c. nomenclature, the A of the ATG translation initiation codon of the EXT1 (NM_000127) or EXT2 (NM_207122) was counted as + 1. For the g. nomenclature, the nucleotide positions were in accordance with hg19.**

**FIGURE 3:**
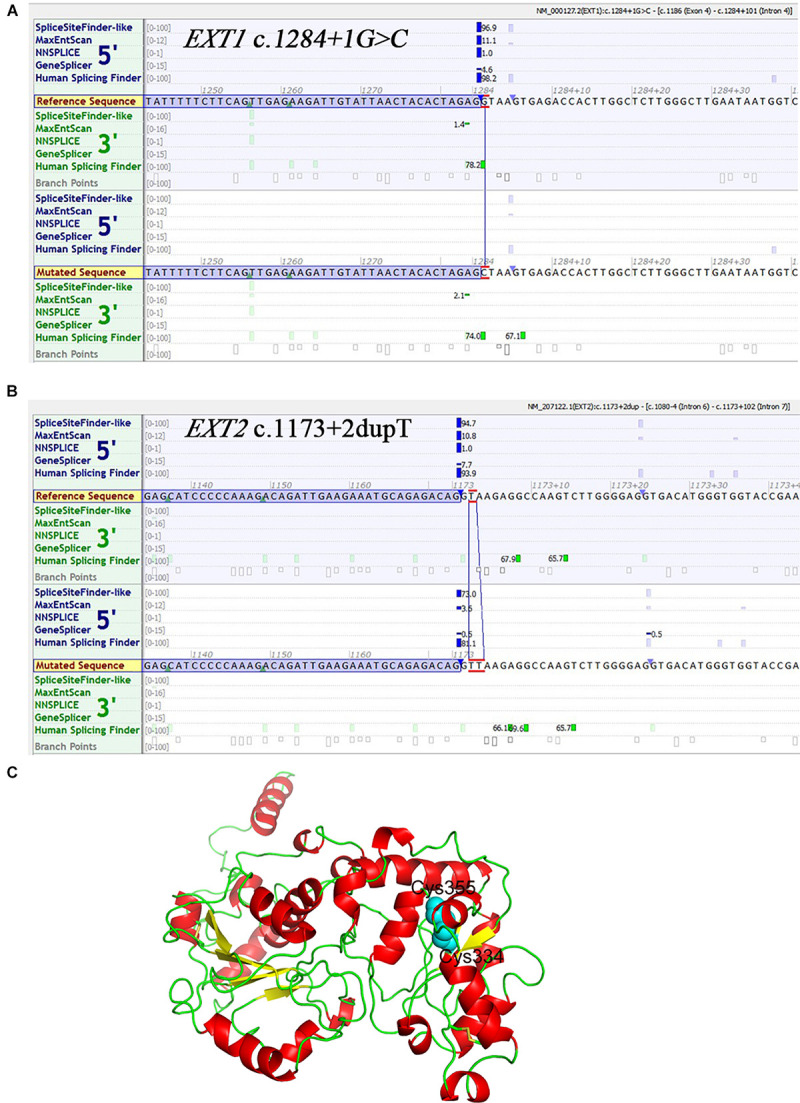
*In silico* analyses with respect to three novel variants. **(A)** Prediction of the potential effect of *EXT1* c.1284 + 1G > C on splicing by Alamut. **(B)** Prediction of the potential effect of *EXT2* c.1173 + 2dupT on splicing by Alamut. **(C)** Structure model of EXT1. The N-terminal region (amino acid residues 29–461) is shown here in cartoon form. Helix, sheet and loop are colored in red, yellow and green, respectively. The side chains of Cys355 and Cys334 are shown as spheres colored in cyan.

Of the 16 unique variants, 12 appear to be loss of function by virtue of their nature, irrespective of whether or not they had been previously described; these included all frameshift (*n* = 5) and non-sense (*n* = 4) variants and the gross duplication and deletion (*n* = 3) variants. Of the two splice site variants identified, *EXT1* c.1284 + 1G > C was also highly likely to be of pathological significance due to its disruption of the canonical 2-bp GT splice donor site (Lin et al., 2019); the Alamut prediction concurred with this assumption (Figure 3A). The pathogenic relevance of the *EXT2* c.1173 + 2dupT variant was not however immediately apparent because it did not alter the canonical 2-bp GT donor splice site. Alamut was therefore employed to predict its impact on splicing. As shown in Figure 3B, all five *in silico* algorithms provided by the Alamut suite under default conditions predicted that the variant allele would exhibit significantly reduced splicing potential as compared to the wild-type allele. We further employed the recently developed SpliceAI (Jaganathan et al., 2019) to predict the impact of the two splice-site variants on splicing and, as previously described (Chen et al., 2020), we focused our analysis exclusively on the Delta scores of donor loss although other scores may provide clues as to the nature of the resulting aberrantly spliced transcripts of splice-altering variants. Both variants were predicted to have a high probability of altering splicing, the Delta scores of donor loss being 0.91 and 0.99 for *EXT1* c.1284 + 1G > C and *EXT2* c.1173 + 2dupT, respectively (Delta scores range from 0 to 1, with 1 indicating the highest probability of altering splicing). As for the two missense variants (both in *EXT1*), c.1019G > A (p.Arg340His) has been reported in > 30 MO patients according to the *EXT1* locus-specific mutation database and was predicted by VarSome to be pathogenic (verdict based on 11 pathogenic predictions from BayesDel_addAF, DANN, DEOGEN2, EIGEN, FATHMM-MKL, M-CAP, MVP, MutationAssessor, MutationTaster, PrimateAI and REVEL vs. 1 benign prediction from SIFT).

The newly described *EXT1* c.1065C > G (p.Cys355Trp) was predicted by VarSome to be “likely pathogenic” (verdict based on 10 pathogenic predictions from BayesDel_addAF, DEOGEN2, FATHMM-MKL, M-CAP, MVP, MutationAssessor, MutationTaster, PrimateAI, REVEL and SIFT vs. 2 benign predictions from DANN and EIGEN). Most importantly, two other missense variants that affected the p.Cys355 site, p.Cys355Arg and p.Cys355Tyr, have previously been identified in MO patients (Jennes et al., 2009; Ishimaru et al., 2016), adding significant weight to our contention that p.Cys355Trp is of pathological relevance. To provide further insight into this question, we performed structural modeling of EXT1. The N-terminal region of EXT1 belongs to the GT47 family, but protein structure information from this domain is so far lacking. We therefore used the latest TrRosetta method on Robetta server (Hiranuma et al., 2020; Yang et al., 2020), a deep learning-based method, to predict the structure of human EXT1. This model shows that the N-terminal part of EXT1 comprises two domains, which adopt a GT-B-like fold. The classical GT-B fold is composed of two distinct N-terminal and C-terminal Rossmann-like domains of six or seven parallel-sheet linked to α-helices, connected by a linker region and an interdomain cleft. This model predicted that the Cys355 is located in the center of the second lobe and can form a disulfide bond with Cys334 (Figure 3C). Therefore, Cys355 probably plays a critical role in stabilizing the protein; substitution of Cys by a larger hydrophobic residue Trp (and other residues) would abolish the disulfide bond, thereby impacting both the stability and enzymatic activity of EXT1.

**FIGURE 4:**
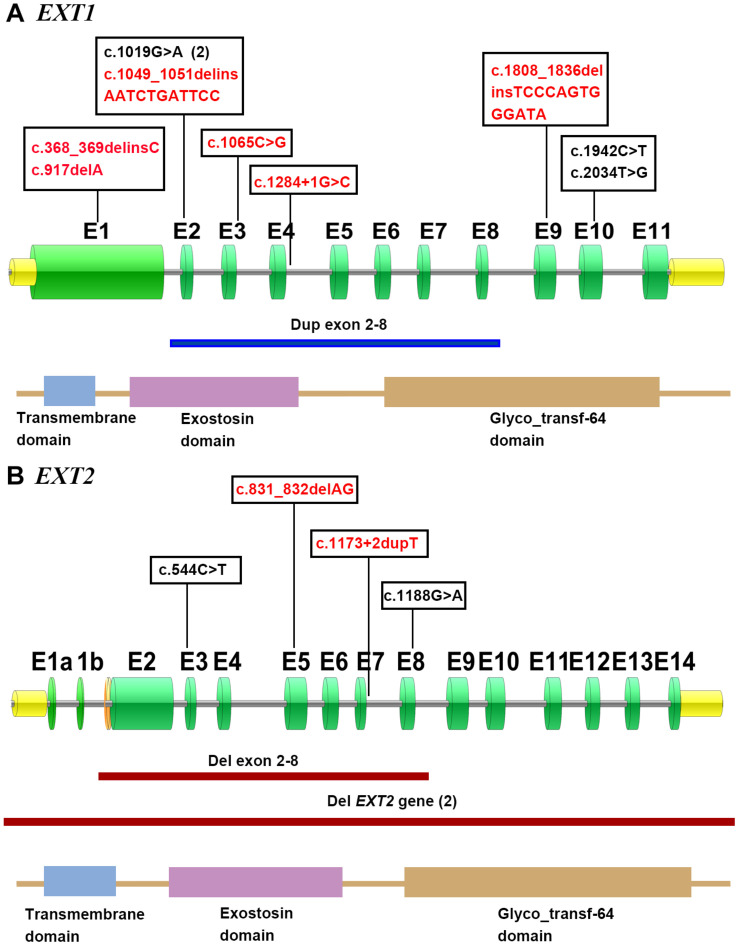
Distribution of the described *EXT1* **(A)** and *EXT2* **(B)** variants in accordance with their respective gene/protein structures. The green boxes denote the numbered exons. Novel mutations are shown in red. The horizontal line indicates the approximate demarcations of the genomic deletions in *EXT2* (red) and duplication in *EXT1* (blue). The protein domains of *EXT1* and *EXT2* are presented at the bottom of each gene depiction, and were derived from the Protein Families (Pfam) database.

In short, pathogenic variants were found in 82% (*n* = 18) of the 22 MO families, with *EXT1* and *EXT2* variants being detected in 50% (11/22) and 32% (7/22) of cases, respectively. Figure 4 illustrates these variants in terms of their locations within their respective gene/protein sequences.

Families 19–22 remained genetically unexplained after whole-exome sequencing (WES) or Sanger sequencing followed by MLPA (Table 2). Karyotyping was further performed on probands of families 19, 20, and 21, showing no chromosome abnormalities (Supplementary Figure S2). This served to rule out the presence of chromosome rearrangements in excess of 5–10 MB in the three cases. Finally, family 19-II-1 was analyzed by WES, the resulting variants of unknown significance are provided in Supplementary Table S1.

### Preimplantation Genetic Testing and Prenatal Diagnosis in 8 MO Families

As MO is an autosomal dominant skeletal disorder, the risk of this disease appearing in the offspring of an affected individual is expected to be approximately 50%. It is therefore extremely important in such cases to be able to provide prenatal diagnosis or PGT with a view to preventing the reoccurrence of MO in the affected families. Of the 18 MO families in which a genetic cause could be unequivocally established, eight sought assistance with this issue (those who were born healthy through the PGT were marked with #, and those who directly received prenatal diagnosis as a fetus were marked with ^∗^ in Figure 1).

Specifically, three couples conceived through *in vitro* fertilization after PGT, a technique whereby a 5-day-old embryo is tested in a laboratory to determine if it carries a particular disease-causing variant. The three couples (Family 4-II-3, Family 5-II-3, and Family 13-II-5) underwent a total of four PGT cycles. Trophectoderm biopsy and PGT-M/PGT-A (PGT for Monogenic defects/PGT for Aneuploidies) were performed on the 27 blastocysts. Twelve (44.4%) of the 27 embryos were found not to harbor any *EXT* mutations by PGT-M whereas 13 of the 27 embryos were found to be euploid by means of PGT-A (Table 3). Three embryos (Embryo-2 of Family 4-II-3, Embryo-6 of Family 5-II-3 and Embryo-1 of Family 13-II-5) found to be both mutative-negative and euploid were used for transfer, all resulting in healthy live births at full term. Moreover, two of the three PGT babies were subjected to prenatal diagnosis, both showing no *EXT1/EXT2* mutations.

**TABLE 3:** Summary of PGT-M/PGT-A results from three families.

Sample no.	PGT cycle	Biopsy time	Embryo	PGT-M result		PGT-A result	Outcome
				
				Halpotype	Sanger Sequencing		
Family 4-II-3	1	Day 5	Embryo-1	Affected	Heterozygote	+ 21	
		Day 5	Embryo-2	Normal	Normal	Euploid	Transfer
		Day 5	Embryo-3	Normal	Normal	−mos(18)(q12.1)	
		Day 5	Embryo-4	Affected	Heterozygote	Euploid	
		Day 5	Embryo-5	Affected	Heterozygote	−mos(6)(q21),-16	
		Day 5	Embryo-6	Normal	Normal	Euploid	
		Day 5	Embryo-7	Affected	Heterozygote	Euploid	
		Day 6	Embryo-8	Normal	Normal	Euploid	
		Day 6	Embryo-9	Affected	Heterozygote	Euploid	
		Day 6	Embryo-10	Normal	Normal	−13	
Family 5-II-3	1	Day 5	Embryo-1	Normal	Normal	−22	
		Day 5	Embryo-2	Affected	Heterozygote	Euploid	
		Day 5	Embryo-3	Normal	Normal	+ (6)(q14.3)	
		Day 5	Embryo-4	Affected	Heterozygote	Euploid	
		Day 5	Embryo-5	Affected	Heterozygote	+ mos(15)	
		Day 6	Embryo-6	Normal	Normal	Multiple chromosome abnormality	
		Day 6	Embryo-7	Maternal Affected Haploidy	Hemizygote	−8, + 22	
	2	Day 5	Embryo-1	Affected	Heterozygote	−15	
		Day 5	Embryo-2	Affected	Heterozygote	+ 22	
		Day 5	Embryo-3	Normal	Normal	−21	
		Day 5	Embryo-4	Affected	Heterozygote	Euploid	
		Day 5	Embryo-5	Affected	Heterozygote	−mos(13)(33%)	
		Day 6	Embryo-6	Normal	Normal	Euploid	Transfer
		Day 6	Embryo-7	Normal	Normal	+ 18	
Family 13-II-5	1	Day 5	Embryo-1	Normal	Normal	Euploid	Transfer
		Day 5	Embryo-2	Affected	Heterozygote	Euploid	
		Day 6	Embryo-3	Affected	Heterozygote	Euploid	

The other five couples (Family 6-II-2, Family 7-II-2, Family 10-II-2, Family 11-II-3, and Family 12-II-2) elected to undergo prenatal diagnosis on the fetus after the mothers had conceived naturally. Genomic DNA, prepared from amniotic fluid cells taken by ultrasound-guided amniocentesis at about 17 weeks of gestation, was used to investigate whether or not the fetus carried the pathogenic variant. The results and outcomes of prenatal diagnosis in the 5 families were summarized in Table 4. Pathogenic mutations were detected in the fetuses in families 10 and 12, and in both cases the pregnant women elected to terminate the pregnancies. Fortunately, no mutations were detected during the second pregnancy of Family 12-II-2.

**TABLE 4:** Summary of the results of prenatal diagnosis in five families.

Sample no.	Number of prenatal diagnoses	Gestation, weeks	Fetus	Methods	Result	Outcome
Family 6-II-2	1	17 + 6 W	Family 6-III-2	Sanger sequencing	Normal	Born healthy
Family 7-II-2	1	17 + 5 W	Family 7-III-2	Sanger sequencing	Normal	Born healthy
Family 10-II-2	1	17 + 4 W	Family 10-III-1	Sanger sequencing	Heterozygote	Labor induction
Family 11-II-3	1	18 + 2 W	Family 11-III-4	MLPA + chromosome microarray analysis	Normal	Born healthy
Family 12-II-2	1	17 + 3 W	Family 12-III-1	Sanger sequencing	Heterozygote	Labor induction
	2	17 W	Family 12-III-2	Sanger sequencing	Normal	Born healthy

In summary, seven babies from the eight MO families that underwent either PGT or prenatal diagnosis were born healthy and no abnormalities were observed in follow-up studies.

## Discussion

In the present study, we describe the analysis of the pathogenetic basis of multiple osteochondromas (MO) in 22 Chinese families. Our findings concur with those published previously in terms of the overall variant detection rate, a preponderance of *EXT1* variants as compared to *EXT2* variants, and the predominance of truncating variants (Santos et al., 2018; Fusco et al., 2019). Nonetheless, several points are worth noting. First, in addition to the previously described large *EXT1* duplication (Su et al., 2015) and two large *EXT2* deletions (Wang et al., 2015), seven novel variants were identified, thereby expanding the mutational spectrum of the *EXT1* and *EXT2* genes in MO. Notably, we identified a novel missense variant, p.Cys355Trp, in one family. The previous report of two other missense variants affecting the same amino acid, together with our newly modeled EXT1 structure, provide firm support for the pathogenicity of p.Cys355Trp. Second, a higher frequency (22.2%) of large deletion or duplication variants was detected in our cohort than in other studies (e.g., 4–9% in White et al., 2004; Signori et al., 2007; Jennes et al., 2008; Pedrini et al., 2011; Delgado et al., 2014), although it should be born in mind that our cohort was relatively small. Third, the absence of a uterus was discovered in a female MO patient in Family 7-III-1. To the best of our knowledge, this is the first time that such a phenotype has been reported in an MO patient. Whether this was due directly to the *EXT1* c.1284 + 1G > C variant identified or to a defect in another unrelated gene(s) is unclear.

Four families (Families 19, 20, 21, and 22) were variant-negative after WES or Sanger sequencing followed by MLPA (Table 2). Families 19, 20, and 21 were additionally analyzed by karyotyping (Supplementary Figure S2). However, MLPA and karyotyping could not readily detect cryptic balanced rearrangements such as inversions potentially affecting the *EXT1* and/or E*XT2* genes; and WES and Sanger sequencing did not target the vast intronic and regulatory regions of the *EXT1* and *EXT2* genes. Moreover, mosaic variants below the mutation detection threshold may be responsible for the mutation-negative probands in Families 20–22 (as well as for some mutation-negative parents in genetically explained families, i.e., parents in Families 1, 2, and 14). Alternatively, these families may be due to variants in the as yet uncloned *EXT3* gene (Le Merrer et al., 1994). Finally, it should be noted that we have tried but failed to perform whole genome sequencing on the probands of Families 19–22 and WES on the probands of Families 20–22 owing to the poor quality and/or insufficient amount of DNA.

Prenatal diagnosis and PGT are the only ways to prevent the reoccurrence of MO in affected families. In this regard, prenatal diagnosis of MO has been previously described in the literature but in each report, at most two cases were studied (Zhao et al., 2009; Zhu et al., 2011; Tang et al., 2013; Bai et al., 2020). Herein, we described the processes and outcomes of prenatal diagnosis in seven families, including five families who received prenatal diagnosis directly, and two families who received prenatal diagnosis to validate PGT results. Our study is thus the largest to date in terms of reported MO patients who received prenatal diagnosis. Moreover, to the best of our knowledge, this is the first time that PGT has been successfully implemented in MO families.

In summary, we identified 16 unique pathogenic *EXT1* and *EXT2* variants (including seven novel ones) in 18 (82%) of 22 Chinese MO families, which has expanded further the mutational spectrum of MO-causing variants in the two genes. The identification of causative variants allowed us to provide genetic counseling to 8 MO patients in terms either of PGT or prenatal diagnosis, thereby preventing the reoccurrence of the disease in the respective affected families. This is the first report of the successful implementation of PGT in affected MO families and also the largest study in terms of reported subjects undergoing prenatal diagnosis.

## Data Availability Statement

The original contributions presented in the study are publicly available. This data can be found here: https://www.ncbi.nlm.nih.gov/bioproject/PRJNA665773.

## Ethics Statement

The studies involving human participants were reviewed and approved by the Ethics Committee of Sun Yat-sen University. Written informed consent to participate in this study was provided by the participants’ legal guardian/next of kin.

## Author Contributions

YW and YL designed the study. YW, LZ, and YJ performed the genetic analysis and bioinformatics evaluations. CX, LD, and YX conducted the clinical and imaging evaluations. YW and J-MC drafted the manuscript. SS assisted with the bioinformatics analysis. CF and DC contributed to data interpretation. All authors contributed to manuscript revision and approved the final manuscript.

## Conflict of Interest

The authors declare that the research was conducted in the absence of any commercial or financial relationships that could be construed as a potential conflict of interest.

## Footnotes

1 http://www.hgmd.cf.ac.uk/ac/search.html

2 https://databases.lovd.nl/shared/genes/EXT1

3 https://databases.lovd.nl/shared/genes/EXT2

4 http://frodo.wi.mit.edu/primer3/

5 https://software.broadinstitute.org/gatk/documentation/tooldocs/current/org_broadinstitute_gatk_tools_walkers_ indels_IndelRealigner.php

6 https://software.broadinstitute.org/gatk/documentation/tooldocs/current/org_broadinstitute_gatk_tools_walkers_bqsr_BaseRecalibrator.php

7 https://software.broadinstitute.org/gatk/documentation/tooldocs/current/org_broadinstitute_gatk_tools_walkers_genotyper_UnifiedGenotyper.php

8 https://www.ncbi.nlm.nih.gov/CCDS/

9 http://www.hgmd.cf.ac.uk/ac/search.html

10 https://varsome.com/

11 https://www.interactive-biosoftware.com/

12 http://robetta.bakerlab.org/

13 https://gnomad.broadinstitute.org/
