# Supplementary material for: EXT1 and EXT2 Variants in 22 Chinese Families With Multiple Osteochondromas: Seven New Variants and Potentiation of Preimplantation Genetic Testing and Prenatal Diagnosis
Source: Front Genet. 2020 Dec 22;11:607838. doi: 10.3389/fgene.2020.607838 (PMC7783290; doi:10.3389/fgene.2020.607838)
Supplement: Supplementary file 1 [file Data_Sheet_1.docx]

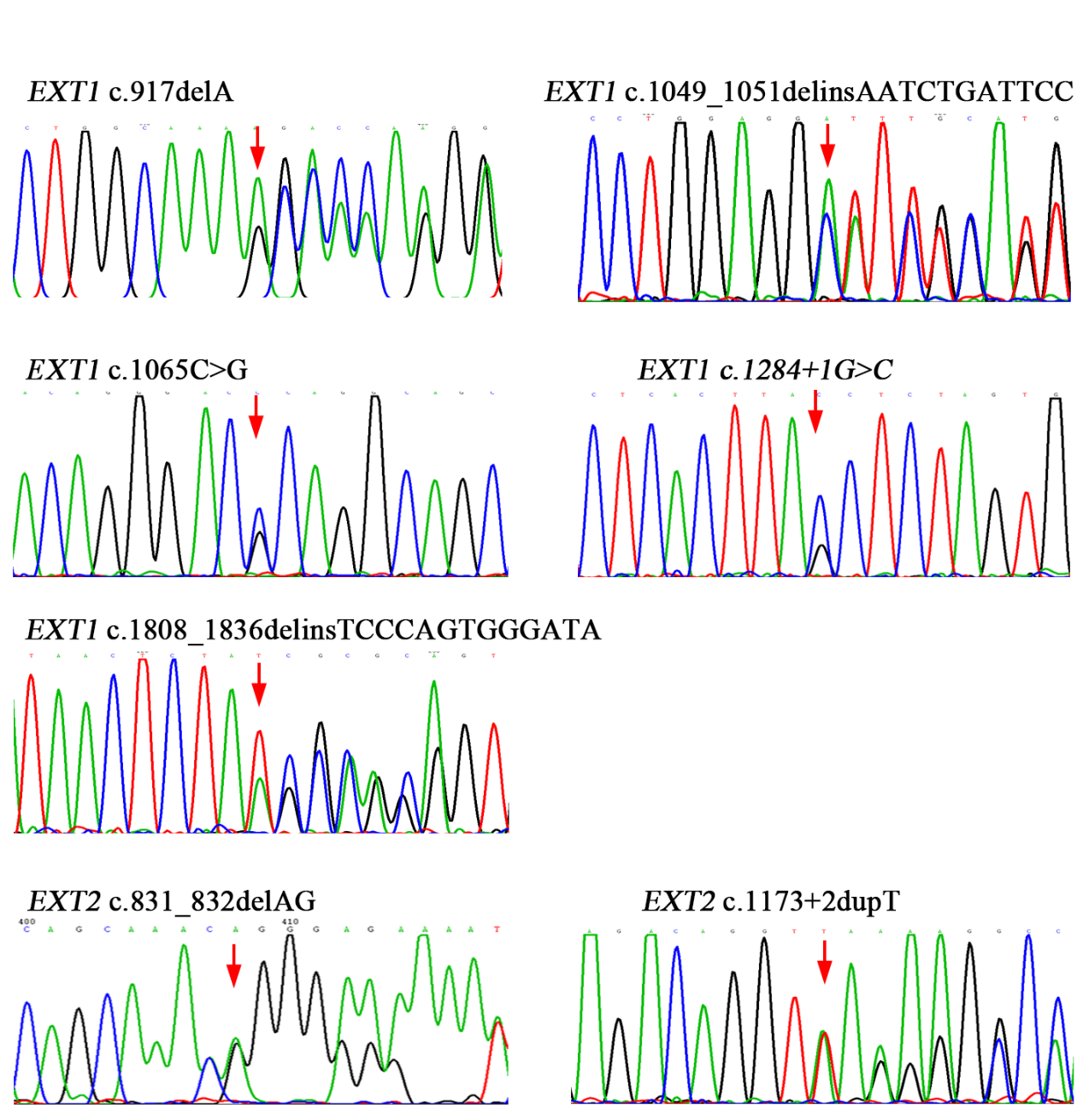


**Supplementary Figure S1.** Sanger sequencing chromatograms for the seven newly described variants.


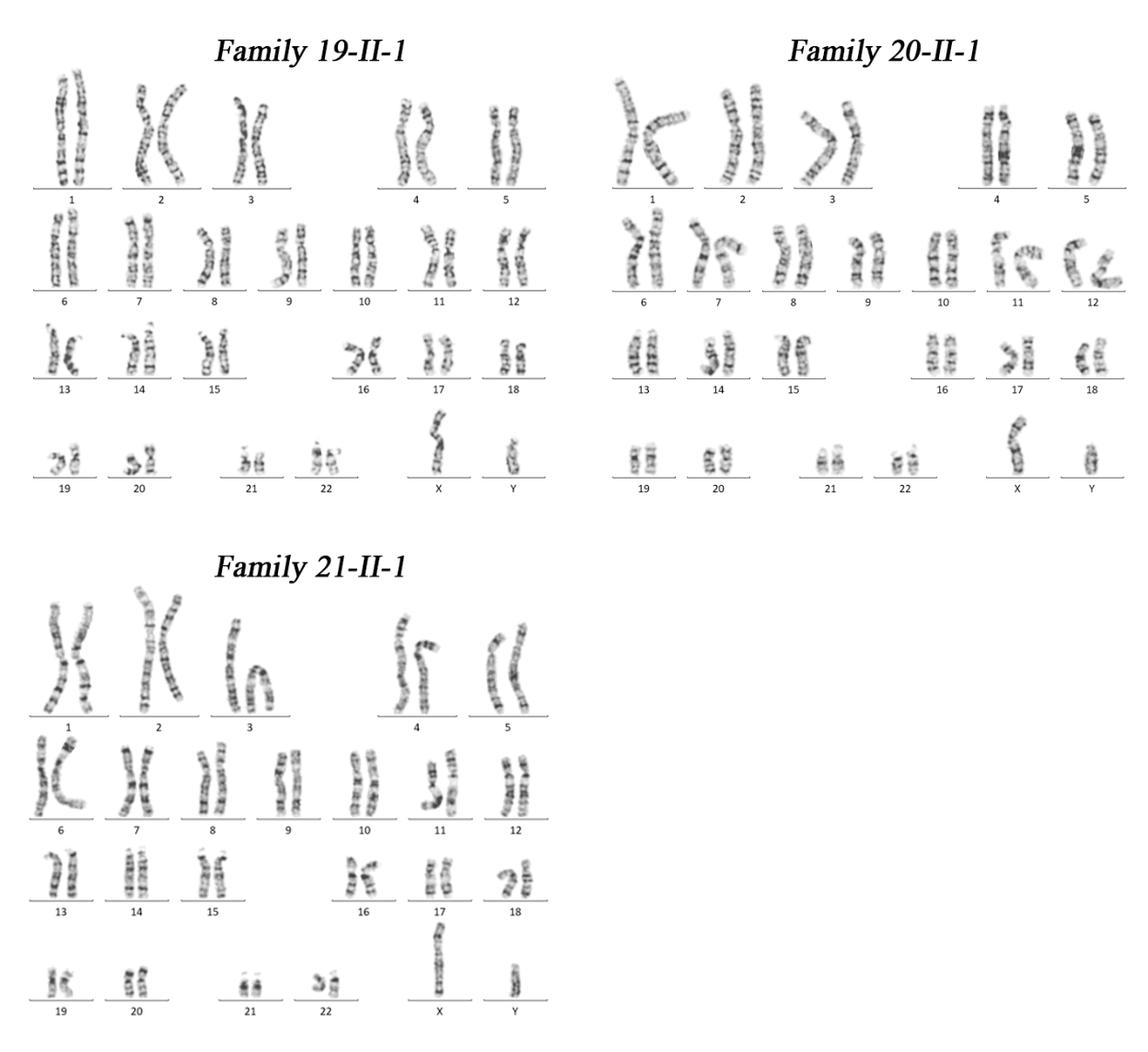


**Supplementary Figure S2.** GTG-banding on karyotypes of Family19-II-1, Family20-II-1 and Family21-II-1.

**Supplementary Table S1.** List of variants of uncertain significance in family 19-II-1 who remained genetically unexplained after whole exome sequencing

| **Gene** | **OMIM number** | **Inheritance** | **Position (hg19)** | **Transcript** | **Nucleotide/Protein change** | **Zygosity** | **Allele Frequency in gnomAD** | **Classification** | **Related disease** |
| --- | --- | --- | --- | --- | --- | --- | --- | --- | --- |
| *ASPM* | 605481 | AR | chr1:197102571 | NM_018136 | c.2328A>T(p.E776D) | Het | 0.003 | VUS | Microcephaly 5, primary |
| *ASPM* | 605481 | AR | chr1:197072778 | NM_018136 | c.5603G>A(p.R1868K) | Het | <0.001 | VUS | Microcephaly 5, primary |
| *CACNA1S* | 114208 | AD | chr1:201016272 | NM_000069 | c.4639C>T(p.R1547W) | Het | 0.001 | VUS | Hypokalemic periodic paralysis, type 1 |
| *CTSA* | 613111 | AR | chr20:44523514 | NM_000308 | c.976C>G(p.L326V) | Het | - | VUS | Galactosialidosis |
| *CTSA* | 613111 | AR | chr20:44526732 | NM_000308 | c.1397C>T(p.A466V) | Het | 0.001 | VUS | Galactosialidosis |
| *ITPR2* | 600144 | AR | chr12:26564324 | NM_002223 | c.7328T>C(p.M2443T) | Het | 0.004 | VUS | Anhidrosis, isolated, with normal sweat glands |
| *LAMA3* | 600805 | AR | chr18: 21357556 | NM_198129 | c.1441G>A (p.E481K) | Het | <0.001 | VUS | Epidermolysis bullosa, generalized atrophic benign/ Epidermolysis bullosa, junctional, Herlitz type/ Laryngoonychocutaneous syndrome |
| *LAMA3* | 600805 | AR | chr18: 21479338 | NM_198129 | c.5923C>T (p.R1975C) | Het | 0.003 | VUS | Epidermolysis bullosa, generalized atrophic benign/ Epidermolysis bullosa, junctional, Herlitz type/ Laryngoonychocutaneous syndrome |
| *MCCC1* | 609010 | AR | chr3:182738001 | NM_020166 | c.1894C>T(p.P632S) | Het | 0.002 | VUS | 3-Methylcrotonyl-CoA carboxylase 1 deficiency |
| *MTO1* | 614667 | AR | chr6:74191938 | NM_001123226 | c.1556A>T(p.D519V) | Het | - | VUS | Combined oxidative phosphorylation deficiency 10 |
| *MTO1* | 614667 | AR | chr6:74210321 | NM_001123226 | c.2062G>A(p.G688R) | Het | <0.001 | VUS | Combined oxidative phosphorylation deficiency 10 |
| *MYO7A* | 276903 | AD/AR | chr11: 76888673 | NM_000260 | c.2266C>T (p.R756W) | Het | <0.001 | VUS | Deafness/ Usher syndrome, type1B |
| *NHLRC1* | 608072 | AR | chr6:18122360 | NM_198586 | c.478T>C (p.C160R) | Het | 0.002 | VUS | Epilepsy, progressive myoclonic 2B |
| *NUP188* | 615587 | AR | chr9: 131730804 | NM_015354 | c.605G>A (p.R202H) | Het | <0.001 | VUS | Sandestig-Stefanova syndrome |
| *PALB2* | 610355 | AR | chr16:23641520 | NM_024675 | c.1955G>A(p.S652N) | Het | 0.001 | VUS | Fanconi anemia |
| *PMFBP1* | 618085 | AR | chr16:72153878 | NM_031293 | c.2879delT(p.L960Qfs*53) | Het | - | LP | Spermatogenic failure 31 |
| *RLBP1* | 180090 | AR/AD | chr15: 89760415 | NM_000326 | c.282delC (p.F95Sfs*24) | Het | 0.001 | LP | Bothnia retinal dystrophy/ Fundus albipunctatus/ Newfoundland rod-cone dystrophy/ Retinitis punctata albescens |
| *TYROBP* | 604142 | AR | chr19:36398118 | NM_001173514 | c.243+2T>C | Het | - | LP | Polycystic lipomembranous osteodysplasia with sclerosing leukoencephalopathy 1 |

Abbreviations: AR, autosomal recessive; AD, autosomal dominant; Het, heterozygosity; VUS, variant of uncertain significance; LP, likely pathogenic
